# Complement, Inflammasome, and Microglial Crosstalk in Glaucoma: From Neurodegeneration to Immune-Based Precision Therapy

**DOI:** 10.3390/life16030368

**Published:** 2026-02-24

**Authors:** Tony Yihao Chen, Na Wu, Xinghuai Sun

**Affiliations:** 1 Department of Ophthalmology and Visual Science, Eye and ENT Hospital, Shanghai Medical College, Fudan University, Shanghai 200032, China; yihaochen23@m.fudan.edu.cn (T.Y.C.); tgf_b@sina.cn (N.W.); 2 NHC Key Laboratory of Myopia, Fudan University, Shanghai 200032, China; 3 Key Laboratory of Myopia, Chinese Academy of Medical Sciences, National Health Commission, Shanghai 200031, China; 4 Shanghai Key Laboratory of Visual Impairment and Restoration, Science and Technology Commission of Shanghai Municipality, Shanghai 200031, China; 5 State Key Laboratory of Medical Neurobiology, Institutes of Brain Science and Collaborative Innovation Center for Brain Science, Fudan University, Shanghai 200032, China

**Keywords:** glaucoma, complement, *NLRP3* inflammasome, microglia, neuroinflammation, retinal ganglion cells, precision medicine

## Abstract

Glaucoma is no longer viewed solely as a pressure-mediated optic neuropathy but as a chronic neurodegenerative disease with a strong immune component. Across experimental models and patient samples, convergent inflammatory circuitry complement activation, *NLRP3* inflammasome signaling, and microglial reactivity emerge as a central driver of retinal ganglion cell (RGC) dysfunction and death. Local complement upregulation (C1q, C3, C5) in the retina and optic nerve head (ONH) promotes aberrant synaptic tagging, phagoptosis, and membrane attack complex stress. In parallel, biomechanical strain, ischemia, mitochondrial damage, and danger-associated molecular patterns prime and activate the *NLRP3* inflammasome in microglia, astrocytes, and ONH cells, leading to caspase-1 activation, IL-1β/IL-18 maturation, and pyroptotic or apoptotic injury. Microglia integrate these cues, shifting from early protective surveillance to chronic maladaptive states that amplify complement and inflammasome outputs. This review synthesizes mechanistic links within the complement *NLRP3* microglia axis, considers systemic and adaptive immune contributions, and proposes a translational framework for immune-based clinical stratification. The literature for this review was identified through searches of PubMed, Web of Science, and Scopus using combinations of the terms ‘glaucoma’, ‘complement’, ‘inflammasome’, ‘*NLRP3*’, ‘microglia’, and ‘neuroinflammation’. Priority was given to recent experimental, translational, and clinical studies. We then evaluate emerging immunomodulatory therapies, complement inhibitors, inflammasome blockers, microglial state reprogrammers, cytokine biologics, and cell-derived immunoregulatory approaches, highlighting biomarkers and trial design needs. An immune systems view of glaucoma enables precision neuroprotection for patients who progress despite controlled intraocular pressure.

## 1. Background

Glaucoma remains a leading cause of irreversible blindness worldwide and is clinically defined by progressive retinal ganglion cell (RGC) degeneration, optic nerve head (ONH) remodeling, and corresponding visual field loss [1]. Although distinct clinical entities are grouped under the umbrella of glaucoma, they share a common final pathway: failure of RGC somas, dendrites, and axons to sustain function under chronic stress [2]. For decades, elevated intraocular pressure (IOP) has been the principal modifiable risk factor, and pressure-lowering remains the cornerstone of treatment [3]. Yet IOP alone does not fully explain disease onset or course. Many individuals develop glaucoma at statistically normal IOP, and a substantial proportion of patients continue to progress despite achieving the target IOP [4]. These observations point to pressure-independent contributors to vascular dysregulation, metabolic aging, genetic susceptibility, and, increasingly, immune-mediated mechanisms that shape neuronal vulnerability and determine progression kinetics.

Over the last two decades, glaucoma research has increasingly converged with paradigms in neurodegenerative disease biology [5]. Rather than viewing inflammation as a late, incidental response to dying neurons, multiple lines of evidence now suggest that immune dysregulation can appear early and actively participate in the degenerative cascade [6]. Transcriptomic surveys of the retina and ONH in animal models reveal enrichment of immune and complement pathways before overt RGC loss, implying that inflammatory priming is not simply secondary to degeneration [7]. Human ocular fluid studies similarly show altered cytokine, chemokine, and complement fragment profiles in glaucoma, even in patients without clinical signs of uveitis or overt ocular inflammation [8]. Imaging and histologic data further support early glial reactivity at sites critical to RGC fate, especially the ONH [9], where biomechanical strain and extracellular matrix remodeling create a uniquely stress-sensitive neuroimmune niche. Together, these findings reposition glaucoma as a chronic, low-grade neuroinflammatory disorder in which immune activity and neurodegeneration are interwoven from the earliest stages.

Central to this reframing is the role of glia as immune effectors. Microglia, astrocytes, and Müller cells are not passive bystanders; they constitute a continuous surveillance network that senses stress signals, removes debris, and secretes trophic mediators [10]. In the healthy retina, these cells maintain synaptic homeostasis, preserve ionic and metabolic stability, and support axonal integrity [11]. Under glaucomatous stress, characterized by IOP fluctuations, ischemia, reactive oxygen species, mitochondrial dysfunction, and altered extracellular matrix tension, glia shift their transcriptional and functional states [12]. Early activation can be adaptive, promoting clearance of damaged organelles and limiting secondary injury. However, when stress is persistent, glia can transition to maladaptive phenotypes characterized by chronic cytokine release, oxidative stress, and excessive phagocytic pruning [13]. The timing and quality of glial state transitions, therefore, represent a key determinant of whether inflammation supports recovery or accelerates RGC attrition.

Among the immune pathways implicated in glaucoma, a recurrent triad emerges across models and human datasets: complement activation, *NLRP3* inflammasome signaling, and microglial reactivity. Complement proteins are produced locally in retina and ONH and become upregulated early in disease, tagging stressed synapses and neuronal elements for elimination while generating pro-inflammatory anaphylatoxins and membrane attack complex stress [14]. In parallel, the *NLRP3* inflammasome serves as a cytosolic danger sensor that integrates mechanical strain, extracellular ATP signaling, mitochondrial reactive oxygen species, and other danger-associated molecular patterns [15]. Its activation drives caspase-1 cleavage and maturation of IL-1β and IL-18, cytokines that potentiate axonal transport failure, synaptic dysfunction, and glial neurotoxicity [16]. Microglia sit at the intersection of these processes: they respond to complement opsonins via CR3, assemble inflammasomes in response to danger cues, and execute phagocytosis and cytokine amplification programs [17]. Importantly, these components form a feed-forward circuit that complements primed microglia and inflammasomes; inflammasome-derived IL-1β induces further complement expression; and complement-tagged targets stimulate microglial engulfment, releasing additional danger signals, helping explain why degeneration may continue even when IOP is controlled.

This immune system view has direct clinical implications. If glaucoma progression is driven by heterogeneous combinations of biomechanical, vascular, metabolic, and immune stressors, then patients cannot be expected to respond uniformly to a single pressure-centered strategy. Immune activity may function as a rate-limiting amplifier in some patients but as a minor epiphenomenon in others. Recognizing immune endotypes—such as complement-dominant synaptopathy, inflammasome-high cytokine toxicity, or microglia-driven phagoptosis could enable earlier detection of aggressive disease and rational selection of mechanism-matched therapies. Moreover, immune pathways are increasingly druggable: complement inhibitors, *NLRP3*/caspase-1 blockers, IL-1 pathway antagonists, and microglial state-reprogramming agents are moving from concept to translational reality in ophthalmology and neurology. In this review, we synthesize current knowledge on the complement–*NLRP3*–microglia axis, consider its integration with systemic immunity and aging, and propose a framework for immunology-informed clinical stratification and immunomodulatory neuroprotection to preserve vision beyond what pressure control alone can achieve.

This review focuses on the microglial and complement–*NLRP3* axes as central inflammatory pathways that we believe drive neurodegeneration in glaucoma. We suggest that the three pathways we have described form a self-reinforcing immune loop linking mechanical and metabolic stressors at the optic nerve head to chronic neuroinflammation, synaptic dysfunction, and retinal ganglion cell degeneration. While we recognize the involvement of other immune and metabolic pathways, this model provides a comprehensive view of mechanisms underlying disease progression beyond the effects of intraocular pressure and informs the formulation of precise strategies to modulate the immune system.

### Inflammatory Pathways Underlying Glaucoma Pathogenesis

Glaucoma has been recognized as a chronic neurodegenerative disease where, alongside other forms of systemic stress, such as mechanical, vascular, and metabolic, immune dysregulation plays a critical role [18]. Although elevated intraocular pressure (IOP) is a risk factor, disease progression continues even after advanced pressure control, suggesting the involvement of additional mechanisms. There is experimental and clinical evidence suggesting that neuroinflammation leads to dysfunction and loss of retinal ganglion cells (RGCs) [19]. In almost all models and patient studies, three interrelated inflammatory regulatory pathways are implicated: complement activation, *NLRP3*, and microglial activation. These pathways are all activated during the early stages of the disease. The pathways demonstrate a self-promoting immune pathway that connects primate-related stressors to neurodegeneration [20].

**Figure 1:**
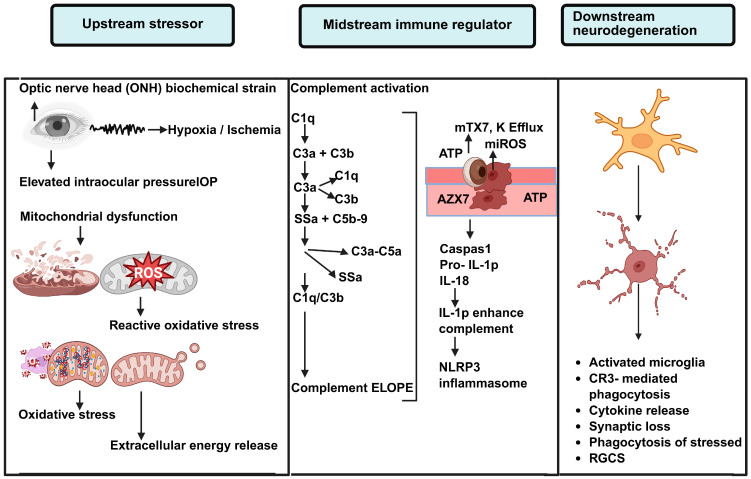
**Immune Mechanisms Linking Glaucoma-Induced Stress to Degeneration.** Schematic representation of the Upstream-Midstream-Downstream immune model facilitating neurodegeneration in glaucoma. Given the Upstream stressors at the optic nerve head (ONH) such as biomechanical stress, hypoxia/ischemia, elevated intraocular pressure (IOP), mitochondrial dysfunction, oxidative stress, and the release of ATP, we refer to these Upstream stressors as Immun Priming. In the midstream (or midstream immune regulatory) phase, local complement activation (C1q, C3, C5) leads to tagging at the synapse and at the cellular level via deposition of C3b, and the anaphylatoxins C3a and C5a are formed, causing an increase in the inflammatory cascade. At the same time, ATP and mitochondrial danger signals produce the efflux of potassium (K^+^), mitochondrial reactive oxygen species (mtROS), and the *NLRP3* inflammatory sequencer, causing activation of caspase 1 and formation of the precursor to IL-1β. IL-1β, produced by the inflammasome, further increases complement expression, creating an inflammatory feedback loop. In the so-called downstream effector stage, activated microglia integrate complement and inflammatory signals, resulting in phagocytosis via CR3, the release of cytokines, synaptic pruning, and phagoptosis of RGCs (or Retinal Ganglion Cells) that are stressed but still viable. This serves to advance neurodegeneration.

At midstream, local complement activation in the retina and optic nerve head (ONH), particularly involving C1q, C3, and C5, leads to erroneous tagging of stressed synapses and neuronal components. While complement supports tissue homeostasis, in glaucoma, the chronic activation of complement drives maladaptive synaptic pruning, phagoptosis of stressed but still viable cells, and membrane attack complex (MAC) associated cellular stress [21]. The complement-derived anaphylatoxins, C3a and C5a, further exacerbate the inflammation by activating resident glial cells. Simultaneously, the *NLRP3* inflammasome integrates upstream danger signals in glaucoma, including biomechanical stress/strain at the ONH, mitochondrial dysfunction, oxidative stress, hypoxia, and extracellular ATP release. After priming and activation, *NLRP3* triggers the caspase-1-dependent maturation of the pro-inflammatory cytokines IL-1β and IL-18. These cytokines cause further synaptic disintegration, disruption of axonal transport, and inflammation within the retina and ONH [22]—see Figure 1.

Microglia act as the central downstream effectors and integrators of these pathways. In response to opsonization and inflammasome stress signaling, microglia initiate programs of phagocytosis and cytokine amplification. While early microglial activation may be a protective response, persistent stress leads to maladaptive phenotypic shifts, particularly to excessive pruning, chronic cytokine release, and oxidative stress [23]. Through the bidirectional interplay between microglia and complement and *NLRP3* signaling, microglia convert episodic stress into a persistent inflammatory response that drives RGC degeneration. Glaucoma pathogenesis framed with the complement–*NLRP3*–microglia triad offers a better understanding of the phenomenon of neurodegeneration ‘on and off the faucet’ with IOP control, and it describes the mechanisms underlying the immune-focused threshold and targeted neuroprotective strategies outlined in the following sections [24].

## 2. Complement Activation in Glaucoma

The complement system is a tightly regulated innate immune cascade that enables rapid recognition and clearance of danger signals. In the eye, complement proteins are delivered not only by the circulation but also synthesized locally by retinal microglia, astrocytes, Müller glia, the vascular endothelium, and stressed neurons, creating an autonomous immune microenvironment [25]. Under physiological conditions, low-level complement activity supports tissue homeostasis by opsonizing apoptotic debris, shaping synaptic refinement during development, and maintaining immune surveillance without overt inflammation [26]. However, complement is an amplification network: once activated, small upstream deviations can generate large downstream inflammatory outputs. Aging, chronic oxidative stress, and metabolic decline shift the complement system toward a “primed” baseline, while glial reactivity and reduced complement regulatory capacity further lower the threshold for maladaptive activation [27]. As a result, glaucomatous stress, whether driven by IOP-related strain, vascular insufficiency, or mitochondrial dysfunction, can push the complement system from a reparative role into a persistent neurotoxic program that accelerates RGC degeneration.

### 2.1. Retinal Complement Physiology and Susceptibility to Dysregulation

Complement activation can be initiated by the classical, lectin, or alternative pathways, each converging on C3 cleavage and the generation of effector molecules such as C3b/iC3b opsonins, the anaphylatoxins C3a/C5a, and the membrane attack complex (MAC) [28]. In the retina, the balance between activation and inhibition is particularly delicate because neurons and synapses require lifelong stability yet operate in an environment characterized by high metabolic flux and photo-oxidative stress [29]. Complement regulators (e.g., CD46, CD55, CD59, factor H) normally restrict indiscriminate opsonization and MAC deposition [28]. With aging and repeated subclinical injury, these regulatory brakes weaken, while complement gene expression rises across glial compartments. Importantly, priming can occur without full activation; increases in complement transcripts and protein deposition can “sensitize” the tissue so that later stressors elicit exaggerated complement responses. In glaucoma, this susceptibility is heightened at the optic nerve head, where biomechanical stretch and extracellular matrix remodeling generate ongoing micro-injuries that sustain complement signaling.

### 2.2. Evidence for Early Complement Upregulation in Glaucoma

Across experimental models, complement induction is among the earliest molecular changes detected in glaucoma. In chronic ocular hypertension and DBA/2J genetic models, C1q and C3 rise before extensive RGC soma loss, paralleling early synaptic dysfunction and axonal transport failure [30]. Complement deposition is prominent in the inner retina and ONH, with microglia and astrocytes showing strong complement signatures in pre-degenerative stages. Human studies reinforce these findings: glaucomatous post-mortem retinas display increased C1q, C3, and MAC, and ocular fluids from patients often contain elevated complement fragments, indicating active local complement turnover [31]. Functionally, complement inhibition or deficiency reduces synapse loss and provides partial neuroprotection in multiple models, supporting a causal role rather than a passive bystander effect. Collectively, these data place complement activation upstream of irreversible neurodegeneration and highlight it as a candidate early therapeutic target.

### 2.3. Complement-Driven Synaptopathy, Phagoptosis, and Inflammatory Amplification

A key mechanism by which the complement system contributes to glaucoma is the aberrant reactivation of developmental synaptic pruning programs. During normal development, C1q and C3 tag weak synapses for microglial removal via CR3-mediated phagocytosis [32]. In glaucoma, stressed RGC synapses and dendrites accumulate complement opsonins and become preferential engulfment targets, producing a synaptopathy that can precede neuronal death and correlate with early electrophysiologic deficits [33]. Complement also promotes phagoptosis, where viable but metabolically stressed RGCs are eliminated after acquiring “eat-me” signals and complement labels, effectively converting reversible stress into permanent cell loss [34]. In parallel, complement fragments C3a and C5a act as potent glial activators, boosting cytokine release, microglial motility, and inflammasome priming [35]. Sublytic MAC deposition adds direct membrane stress and oxidative signaling, further amplifying inflammation [36]. Because the complement system can both tag targets and intensify inflammatory tone, it functions as a dual-action driver of glaucoma progression removing synaptic/neuronal structures while simultaneously escalating local immune reactivity.

### 2.4. NLRP3 Inflammasome Signaling in Glaucoma

The *NLRP3* inflammasome activates inflammatory components in response to cytoplasmic stressors, such as proteotoxic stressors [37]. Within the complex *NLRP3* signaling cascade, the *NLRP3* inflammasome facilitates a cytotoxic response by promoting the release of pro-inflammatory cytokines. Though glaucoma is characterized by chronic biomechanical strain, retina, optic nerve head (ONH) tissue) In metabolic deficiency, *NLRP3* provides the mechanistic link from “danger signals” to the release of neurotoxic cytokines [38]. The “classical” *NLRP3* consists of the *NLRP3* sensor, *NLRP3*, an apoptosis-activating signal containing [ASC], and pro-caspase-1. Upon assembly, caspase-1 activation occurs, cleaving pro-IL-1β and pro-IL-18 to release IL-1β and IL-18, and promoting gasdermin (GSDM)-mediated pore formation in the cell outer membrane, which may lead to pyroptotic cell death [39]. *NLRP3* signaling does not operate as a binary on–off process but instead requires a priming phase that establishes inflammasome competence, followed by episodic activation in response to metabolic or mechanical stress. This sustained signaling is likely a major driver of pressure-independent retinal ganglion cell (RGC) degeneration, due to the condition’s sustained priming and episodic activation. This sustained signaling from RGC inflammation is likely responsible for the inflammation observed across the axis in response to the ONH, a secondary inflammatory response.

**Figure 2:**
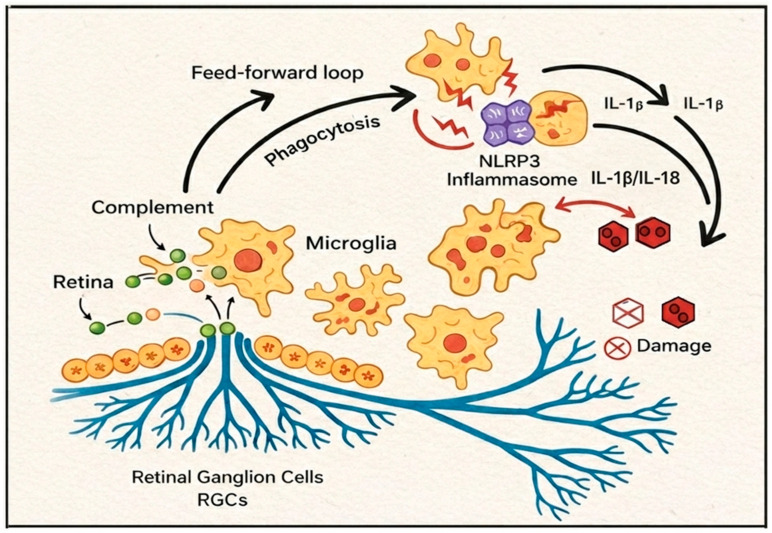
**Complement–*****NLRP3*****–Microglia Axis in the Retina and Optic Nerve Head.** Illustrated Overview of the Complement-*NLRP3*-Microglia SELF-AMPLIFYING INFLAMMATORY CIRCUIT IN NEURODEGENERATION PATTERNS IN GLAUCOMA. Neurodegeneration and the stress of glaucoma result in the activation of the complement system and the onset of neuroinflammation and the resulting neurodegeneration of glaucoma. Stress-induced opsonization of synapses and surrounding neuronal structures occurs. Opsonized-activated microglia, in addition to complement phagocytosis, release DAMPs (danger-associated molecular patterns) which serve to further amplify the signal propagation of innate immune system activation. Biomechanical strain and energy (ATP) and metabolically driven stress on activated microglia also promote *NLRP3* lnlamsome activation/caspase-1 dependent maturation of the pro-inflammatory cytokines IL-1β and IL-18. Microglia activation and pro-inflammatory cytokine IL-1β and IL-18 release further amplify the neuroinflammation complement cascade resulting in cellular degeneration and neural cellular, especially retinal ganglion cell (RGC) layer cell death.

Activation of *NLRP3* occurs in a two-signal manner. The first part is the Priming step (Signal 1), whereby *NLRP3* and pro-IL-1β transcription is upregulated via NF-κB activation (Figure 2). This is triggered by stimuli such as Toll-like receptor ligands, cytokines like *TNF*-α, and complement anaphylatoxins [40]. In tissues affected by glaucoma, chronic glial reactivity and para-inflammation can induce an early form of this step, creating a ‘immune baseline sensitization’ in the tissues. To move on to the next step (Signal 2), activations trigger the assembly of the *NLRP3* inflammasome in the presence of a plethora of intracellular disturbances, including, but not limited to, the P2X7 receptor signaling, efflux of K^+^, destabilization of lysosomes, presence of reactive oxygen species (mtROS), oxidized mitochondrial DNA, and injuries within the cytoskeleton. It should be noted that activation and priming do not have to be executed and completed in a routine manner. The primed retina can be in a latent state and be triggered to rapidly assemble the inflammasome when there are bursts of mechanical or metabolic stress [41]. The ONH is particularly susceptible to these events due to strain-activated DAMP release, whereby the activations can be repeatedly triggered over the course of a priming inflammation state.

Patterns of inflammasome pathway engagement are observed across human samples and preclinical glaucoma models. In vivo models of glaucoma, including chronic ocular hypertension, ischemia–reperfusion injury, and genetic glaucoma, typically show increases in *NLRP3*, ASC, and cleaved caspase-1, often well before there is significant loss of RGC somas [42]. Microglia and astrocytes exhibit these same changes, as do some cells in the ONH, indicating an inflammasome response across multiple tissues. At the same time, human glaucomatous retinas and ONHs show elevated *NLRP3* transcription and protein expression, as well as heightened levels of the pro-inflammatory cytokines IL-1β and IL-18 in aqueous and vitreous samples [43]. Evidence from human studies shows that loss of inflammatory activity via *NLRP3* and caspase-1, and the use of selective inhibitors that preserve RGC endogenous neuroprotective axonal functions, attenuate IL-1β/IL-18 release [44]. Thus, *NLRP3* signaling is not a reliable injury marker. It is an active participant in the inflammatory neurodegenerative process.

The glaucomatous microenvironment has several well-described *NLRP3* triggers. In the lamina cribrosa and ONH, mechanical strain disturbs the axonal cytoskeleton and extracellular matrix, then releases ATP and DAMPs that potently activate P2X7-*NLRP3* signaling [45]. Vascular dysregulation and hypoperfusion introduce intermittent hypoxia that facilitates a shift in retinal metabolism to one that is oxidative, mtROS, and hypoxia state, which is a potent activator of the inflammasome. Mitochondrial dysfunction further exacerbates the deficits observed in aging RGCs, due to increased cytosolic leakage of oxidized mtDNA and sustained active *NLRP3* even under controlled IOP [46]. *NLRP3* is primarily responsible for exacerbating the inflammatory response through the release of IL-1 and IL-18, which compromises synaptic integrity and impairs axonal transport, and makes astrocytes and microglia neurotoxic [47]. IL-18 exotic neuroinflammatory response. Additionally, pore formation in glia and possibly neurons, and the direct cell-loss pathway driven by inflammation, are mediated by pyroptosis, which is also initiated by *NLRP3*. *NLRP3* has an overall integration into immune feed-circuit that enables sustained progression over time. *NLRP3* Inflammasome Signaling in Glaucoma is explained in detail in Figure 2.

## 3. ATP Signaling in Glaucoma

The pathophysiology of glaucoma is diverse, and the ATP imbalance reflects distinct cellular processes. Intra- and extracellular ATP levels differ in the context of glaucoma-associated neuroinflammation [48]. While the intracellular component reflects bioenergetic failure caused by ATP depletion, the extracellular portion of ATP reflects inflammation. The different ATP pools provide context for the imbalance and the conversion of stress into a neuroinflammatory state. The persistent stressors of glaucoma, including intraocular pressure, ischemia, and oxidative stress, cause retinal ganglion cell (RGC) and glial cell bioenergetic failure through downregulation of oxidative phosphorylation, ATP depletion, and elevated mitochondrial dysfunction [49]. ATP depletion contributes to the failure of cellular processes such as axonal transport, synaptic maintenance, and ionic homeostasis. This sets off a cascade of events that increase cellular vulnerability to degeneration and reflect impaired cellular energy metabolism rather than an inflammatory signal per se. Juxtaposed with the exception of activated microglia, all other cells activated by the A1 phenotype result from cell death and the release of ATP as a pro-inflammatory signaling molecule [50]. ATP is a signaling molecule involved in many important biological processes, but in the case of glaucoma, it serves mainly as a signaling molecule of the disease process. ATP is released by neurons and glia that are metabolically stressed and damaged, as well as strained mechanically. This is especially true in the optic nerve head, the area of greatest concentration of ATP. ATP is released and impacts purinergic receptors, especially the P2X7 receptor of glia and microglia, in the extracellular space. Activation of the purinergic P2X7 receptor triggers a series of intracellular processes that include potassium efflux and calcium influx, as well as the generation of reactive oxygen species and the destabilization of lysosomes, all of which are essential for the activation of the *NLRP3* inflammasome [51]. Other important functions of ATP in the inflammatory process are the crucial mechanisms linking inflammasomes and the activation of the complement immune system. Local ATP release results from synaptic tagging and microglial phagocytosis, both of which are mediated by the complement system. Activation of the purinergic P2X7 receptor, mediated by ATP, enhances the assembly of the inflammasome and the maturation of cytokines, including IL-1β and IL-18, through activation of caspase-1. These interleukins further activate and increase expression of the complement system, exposing another loop within the inflammatory process [52]. In summary, it can be stated that with metabolic deficiency, there is always a depletion of intracellular ATP; in contrast, the presence of extracellular ATP signifies a pathological process, in this case signaling through the complement system to enhance *NLRP3* and microglial mechanisms of inflammation, thereby causing synaptic dysfunction, phagocytosis of viable but stressed neurons, and the degeneration of retinal ganglion cells [53].

RGCs are vulnerable to intracellular ATP depletion. ATP depletion is associated with impaired mitochondrial function, oxidative stress, and disrupted axonal transport and occurs in glaucoma. In this context, NVAD+ plays an important role in neuronal energy metabolism and stress adaptation. NVAD+ is a critical cofactor for mitochondrial oxidative phosphorylation, redox homeostasis, and DNA damage repair [54]. Availability of NVAD+ is linked to neurodegeneration and increasing age. Animal studies in glaucoma models show that NAD+ precursors, especially nicotinamide, enhance mitochondrial function, maintain intracellular ATP, and improve RGC survival, even under mechanical or pressure-related stress. NAD+ also limits mitochondrial production of reactive oxygen species and reduces ATP-induced granular stress, which, in turn, decreases microglial activation via the ATP-P2X7 and *NLRP3* signaling pathways, which are downstream of the inflammasome [55]. These effects of NAD+ enhance energy homeostasis and inflammatory control, thus acting as an upstream regulator of the crosstalk among complement, inflammasome, microglia, and are treatable as elements of precise neuroprotective strategies in glaucoma [56].

## 4. NLRP3 Inflammasome and Microglial Signaling in Glaucoma

Complement activation, *NLRP3* signaling, and microglial reactivity form interconnected components of an inflammatory network in glaucoma, even though they are often studied separately. Their interdependence seems to indicate that they form a positive feedback loop, rather than a linear cascade. This interdependence could bring together upstream biomechanical and metabolic stress, sustained neuroinflammation, and the progressive degeneration of retinal ganglion cells (RGCs) [38]. Immune activation is a resultant response of elevated and fluctuating intraocular pressure, biomechanical strains at the optic nerve head (ONH), ischemia, mitochondrial dysfunction, oxidative stress, extracellular ATP release, and the sequence of events that unfold at the retinal and ONH tissue during glaucoma. While these do not directly eliminate RGCs, they do, however, trigger the immune system. At this point, complement activation and *NLRP3* signaling are the main stress signal amplifiers [48]. Complement system components (C1q, C3, C5) are upregulated at stressed neuronal membranes and synapses, and the anaphylatoxins (C3a, C5a) activate glial cells and increase the expression of inflammatory proteins. At the same time, *NLRP3*, as a result of ATP–P2X7 signaling, mitochondrial ROS formation, potassium efflux, and the presence of oxidized mtDNA, drives the activation of the inflammatory caspase-1 and the maturation of the inflammatory cytokines IL-1β and IL-18. Notably, the pathways described above operate bidirectionally. NF-kB-dependent transcriptional mechanisms may work to activate the components of the *NLRP3* inflammasome by priming inflammasome assembly. On the other hand, the IL-1β class of inflammasomes is responsible for further increasing the expression of genes associated with the complement system, leading to increased activity of the complement system. Even without increases in IOP, the sustained, self-priming states of inflammation can be maintained by the processes described above. At the level of the downstream effectors, microglia respond to complement [51].

The phagocytic activity of microglial CR3 is engaged by the complement-tagged synapses and neurons, and the activity of IL-1β and IL-18 increases the migration of microglia, the release of cytokines, and the generation of reactive oxygen species. While the early activation of microglia may facilitate the clearance of debris and the surveillance of tissue, its prolonged activation leads to pathologic changes and the development of maladaptive phenotypes, including excessive synaptic pruning, phagoptosis of stressed but viable RGCs, and sustained inflammatory amplification [57]. Altogether, the microglia–complement–*NLRP3* axis operates as a closed-loop system, converting episodic mechanical and/or metabolic stress into progressive stress-related neurodegeneration that is independent of intraocular pressure. It is crucial to appreciate the integrated crosstalk among these pathways to identify precise therapeutic targets and to develop immune-based strategies that disrupt the pathological amplification process while preserving the early beneficial immune response [58].

### 4.1. Microglia Versus Astrocytes

Microglia and astrocytes play distinct and complementary roles in responding to inflammation and stress in glaucoma. Microglia serve as the first line of immune response, responding rapidly through phagocytosis and complement-mediated opsonization. This includes the release of certain cytokines and activating of the *NLRP3* inflammasome [59]. Activated microglia retain synapse loss and degeneration of retinal ganglion cells through their direct interaction with complement receptor, synaptic phagocytosis, and pruning. Subsequently, astrocytes play a counterintuitive role by modulating the tissue. During astrocyte reactivity, there is an adjustment in extracellular ion equilibrium and the removal of glutamate, which also affects the metabolic support of the tissue and the blood-retinal barrier. Astrocytes also modulate neuronal vulnerability and glial cell activation. Compared with microglia, which have an abundant capacity for phagocytosis, astrocytes have very little, which contributes to their immune-priming function. Therefore, microglia are key players in immune-driven neurodegeneration, whereas astrocytes modulate the environmental factors in which neurotissue inflammation occurs [60] (Table 1).

### 4.2. Integration: The Complement–NLRP3–Microglia Axis as a Feed-Forward Circuit

Microglia-associated neuroinflammation may indirectly influence aqueous outflow and biomechanical susceptibility, but evidence for direct regulation of IOP remains limited. Complement fragments sensitize microglia and also enhance transcription of *NLRP3*. Moreover, isolated IL-1β from microglial inflammasome is a complement activator. Complement-bound synapses also trigger DAMPs to re-activate inflammasomes, and through ATP, synapses cause fast-acting neuroinflammation. This explains disease progression and IOP.

## 5. Systemic and Adaptive Immune Contributions Beyond the Triad

While complement, *NLRP3* signaling, and microglial activation form the core circuit of the retina’s innate immune system, glaucoma develops within a larger context of the body’s defenses that go beyond the eye. The retina and ONH are not fully immune-isolated compartments; they are subject to circulating inflammatory signals and age-related changes in the immune system, and to systemic metabolic or vascular triggers that alter local immune responses. Clinical and experimental studies are showing that peripheral immune responses can trigger or prolong inflammation in the eye, and that inflammation can act independently of IOP to alter the course of disease. This broadened frame of reference helps explain the wide variability in disease course; in particular, normal-tension glaucoma, in which dysregulation of vascular flow and a systemic inflammatory state may be more important than intraocular inflammation. Critically, these systemic influences do not supplant local mechanisms; rather, they enhance local retinal mechanisms, thereby determining whether complement–inflammasome–microglia activity is adaptive or transitions into chronic neurotoxicity.

### 5.1. Peripheral Immune Trafficking and Barrier Modulation

While none of the complications of glaucoma include the obvious leukocytic infiltration noted in uveitis, low-grade peripheral immune involvement has been supported by multiple studies. According to some studies of animal models, the upregulation of some chemokines and the minor weakening of the blood retinal barrier and blood optic nerve barrier allow for the intermittent enclosure, or perivascular accumulation of monocytes and T cells within the optic nerve head (ONH) and inner retina [68]. Factors such as chronic complement activation and cytokines from inflammasomes that permeate the endothelium and modify adhesion molecules within the bloodstream can all facilitate such trafficking. Even when cells that infiltrate the body are few in number, their effects can be substantial because they secrete cytokines, present diverse antigens, and modify the programs of microglial cells in the local environment. For instance, circulating monocytes can acquire a microglial phenotype but are more likely to retain their pro-inflammatory bias, which reinforces local tissue complement production and IL-1β signaling [69]. Entry of T-cells, even at low levels, can further skew the immune response in the retina, promoting sustained antigen presentation and glial activation [70]. These relaxations suggest that in patients with systemic inflammatory comorbidities, immune trafficking, along with narrow barrier modulation, acts as a “silent amplifier” of glaucomatous neuroinflammation.

### 5.2. Autoantibodies, Adaptive Immunity, and Immune Memory

**Figure 3:**
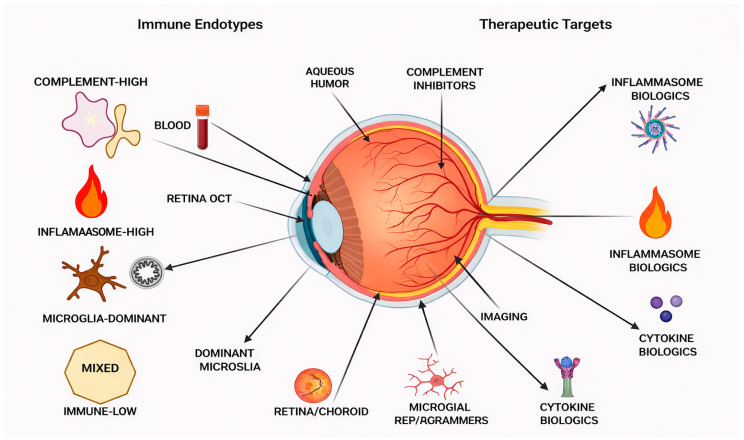
**Immune-Based Clinical Stratification and Therapeutic Targets in Glaucoma.** Conceptual framework for immune-informed clinical stratification in glaucoma. Different immune endotypes with candidate biomarkers from the eye’s aqueous humor, blood, retinal imaging, and optical coherence tomography (OCT) complement-high, inflammasome-high, microglia-dominant, mixed, and immune-low are illustrated. Potential therapeutic targets (complement inhibitors, inflammasome and cytokine biologic, microglial state reprogramming) are presented. This framework shows that the combination of immune biomarkers and ocular imaging data may allow neuroprotection to be tailored beyond the management of intraocular pressure.

Reports on adaptive immune signatures in glaucoma, particularly autoantibody responses to heat shock proteins (HSPs), retinal antigens, and myelin-associated autoantigens, have appeared over the years, including the first reports [71]. These antibodies may arise from the first contact of an antigen(s) that arise from the incident axonal stress and/or synaptic injury, after which immune memory, of which the form(s) are not clear, can become re-engaged and lead to an enhanced cycle of injury and degeneration. A feature of experimental autoimmune glaucoma is the ability to reproduce some aspects of glaucomatous RGC loss after systemic immune priming with retinal antigens or HSPs, suggesting that these factors are causative in a subset of glaucomas [51]. In these cohorts, some patients with higher HSP antibodies and T cells that are active showed faster progression, while some patients showed no changes, which may reflect the heterogeneity of the case(s) in the cohort or the adaptive immune system [72]. Mechanistically, adaptive immunity may facilitate the pathway through at least but not limited to (1) synaptic tagging that is enhanced through the irreversibility of the pathway via complement that is activated through antibodies; (2) the phagocytosis and decreased cytokine activity on microglia via Fc receptor signaling; (3) the cytokines from T cells that are specific to the targets and the maintenance of the inflammasome priming that is in an active primed state Figure 3. Autoantibodies may, in some if not all instances, reflect a sequencing immune identification of antigens that are released, but the presence of these antibodies may not be of pathogenic origin. However, the presence of autoantibodies, which have been documented in these studies, give support to the idea of systemic immune priming, which may in some individuals lead to a lack of local innate mechanisms of an increase in chronic dysregulation.

### 5.3. Aging, Trained Immunity, Metabolic Inflammation, and Neurovascular Coupling

The strongest risk for glaucoma is linked to age. Aging also causes changes to both systemic and retinal immunity. In particular, innate immune cells such as microglia experience what is called ‘inflammaging’ [73]. This involves an increase to baselines in NF-κB activity, complement expression, and activation to mitochondrial DAMPs and increases their sensitivity to activation through *NLRP3* and complement cascades, which can cause prolonged inflammatory output over less severe stressors [74]. Meanwhile, even though microglia may already be in a hyper-responsive state due to trained immunity from repeated systemic shocks, they can become even more reactive from chronic infections, inflammatory diseases, and environmental stress. Additionally, metabolic diseases such as diabetes, obesity, and dyslipidemia contribute to sustained oxidative and glycation stress, which impairs RGC mitochondrial function, and primes inflammasome activation and complement dysregulation for further related systemic change [46]. Neurovascular factors are also particularly important in normal-tension glaucoma. Impaired autoregulation, nocturnal hypotension, and microvascular insufficiency cause repeated episodes of ROS and *NLRP3* activation hvia Hypoxia and ROS bursts, alongside systemic complement dysregulation. These also cause fragmented complement to impair endothelial function and increase inflammatory cell adhesion, which recruits immune cells to the site of exacerbated vascular dysfunction. Consequently, aging, metabolic inflammation, trained immunity, and vascular instability interact to create an environment that strongly favors an exacerbated inflammatory response, thereby perpetuating neurodegeneration even in the context of controlled IOP.

## 6. Clinical Stratification Through an Immunology Lens

OCT and visual field staging reveal structural damage with a considerable delay in the causal pathway. Immune-based profiling enables the identification of patients with rapid disease progression, facilitating targeted immunomodulation. Immune-high phenotypes may have a preferential response to the termination of complement activity or to the blockade of inflammasomes. Current glaucoma management is based on stratifying disease by OCT RNFL/GCC cross-sections, ONH morphology, and standard automated perimetry function tests [75]. These results remain critical for diagnosis and subsequent evaluation; however, they are late-stage outputs of cumulative injuries rather than primary drivers of degeneration. For a measurable thinning of RGCs and synapses, there is a large loss with little recovery. This becomes especially vexing for patients with progression at apparently well-controlled IOP, as pressure-centric escalation becomes of limited benefit. An immunology-informed framework proposes that distinct inflammatory endotypes, defined by complement-mediated synaptopathy, inflammasome-driven cytokine toxicity, and maladaptive microglial states, influence both susceptibility and the rate of progression [76]. That suggests a need for clinical staging based on immune activity to (1) separate high-risk progressors from the rest, (2) divide patients into distinct mechanistic subgroups currently grouped together, (3) assign patients to targeted immunomodulatory neuroprotection. This kind of stratification is not proposed as a replacement for IOP management, but rather as a complement, introducing a second axis to capture biology inaccessible to conventional imaging.

### 6.1. Why Immune Stratification Is Needed

Glaucoma is among the most heterogeneous ocular diseases. Patients can have the same IOP exposure, the same optic nerve anatomy, and the same baseline OCT findings yet exhibit markedly different disease trajectories. Their trajectories can range from a slow decline spanning decades to a rapid functional collapse within a short period [77]. The classic risk factors, such as patient age, corneal thickness, family history, and vascular status, can only partially explain the observed disease trajectories. Immune “set points” is a plausible suggestion. Some patients may present with a complement-primed retina, an inflammasome with heightened sensitivity, and microglia that readily switch to neurotoxic phenotypes. These patients can have low IOP and experience significant synapse loss and RGC death, as the inflammatory processes can endure even with minimal injuries. In contrast, patients with low immune responses may have clinical or vascular disease progression and derive little benefit from systemic or intraocular immunotherapy. The lack of immune stratification makes clinical trials with immune interventions prone to dilution among responders, which can lead to failure. The clinical side can also suffer from this lack of immune stratification, as immunodeficient patients are exposed to unnecessary risk from immunomodulatory therapy. Immune stratification is necessary for precise neuroprotection, trial enrichment of probable responders, and to explain the clinic’s pressure-independent progression in patients.

### 6.2. Candidate Biomarker Categories

Measurable, reproducible, and mechanistically interpretable biomarkers, if available, can facilitate the construction of a reliable stratification system. One potential area of predictive modeling is ocular fluid profiling. During scheduled surgical procedures, researchers can obtain aqueous humor samples and quantify the immune profiles in both cytokines (e.g., *IL-1β*, *TNF-α*, *IL-6*), chemokines, and complements (e.g., C3a, C5a, iC3b) to record the immune tone in proximity to the retina and ONH (optic nerve head) in real time. Imaging-linked inflammation is another potential domain [78]. Hyperreflective foci can be spotted on structure-light OCT, and microglial-like cell aggregates can be identified in Adaptive Optics with downstream molecular imaging. Synaptic suppression may indicate upstream microglial activation and complement-mediated suppression of neural circuitry [79]. A third domain, systemic immune signatures, captures peripheral cytokines, and transcriptomic panels can quantify priming of the immune system that is both peripheral and biased toward the retina. A fourth system employs predictive modeling of polygenic and genetic data related to specific pathways. Complement regulatory genes, inflammasomes, and microglial-modulating genes could facilitate the prediction of the immune-dominant phenotype. Finally, a set of immune functioning stress tests (e.g., ex vivo assays, monocyte models, or microglial models) may yield personalized parameters of immune reactivity.

### 6.3. A Practical Immune-Based Stratification Framework

If Immune measures were integrated with traditional progression tracking, it would create a straightforward clinical system, eliminating the need for completely new systems. First, patients would be longitudinally segmented based on the slope of structural/functional progression using OCT and visual field data. Next, we would estimate local complement and IL-1β–axis activity by measuring an immune aqueous panel (obtained opportunistically from cataract or glaucoma surgeries or from trial settings). Third, an integrated systemic inflammation score from blood biomarkers would determine patients with high peripheral priming. Finally, targeted genotyping could further refine complement or inflammasome-dominant phenotypes. Endotypes include (1) complement-dominant synaptopathy (high ocular complement and early deficit dysfunction), (2) inflammasome-dominant cytokine toxicity (high IL-1β/IL-18 signature), (3) microglia-dominant with maladaptive activation (via imaging proxies and innate signature elevation), (4) immune-metabolic disease mixed (high immune and mitochondrial/vascular stress), (5) immune-low glaucoma (pressure/vascular drive with faint immune signature). Each stratification implies a distinct treatment and rationale for matching patients to mechanism-driven immunomodulatory trials (Table 2).

## 7. Immunomodulatory Therapies: From Mechanism to Clinic

The increasing recognition that immune dysregulation can cause RGC loss has drawn attention to glaucoma therapeutics centered on immunomodulatory neuroprotection [80]. In contrast to pressure-lowering agents, immune-targeted therapies aim to mitigate neurodegeneration at the synaptic level by modulating synaptic tagging and pruning, amplifying danger signals, mitigating chronic cytokine toxicity, and reducing inappropriate glial phagocytosis, even when IOP is controlled. Translational readiness is aided by two factors: first, the overlap of immune dysregulation in glaucoma with that in other retinal and neurological diseases, which already have drug platforms; and second, the complement *NLRP3* microglia triad, which provides distinct, stage-linked targets. However, context-dependent immune responses pose a risk, as the same pathways that are protective in the early stages of a disease can become detrimental if engaged for prolonged periods. Therefore, therapeutic success is expected to arise from combining mechanism-matched drugs with immune endotyping to determine appropriate treatment windows, using collateral biomarkers to confirm target engagement, and targeting the most relevant targets of immunoregulatory therapeutic strategies. Below, we summarize three strategy classes that map directly onto the triad and its extensions, emphasizing the potential for their immediate deployment in clinical settings.

### 7.1. Complement-Targeted Neuroprotection

Complement inhibition is appealing given the early complement upregulation during the synaptic stage, which also fuels the inflammatory cascade. Early intervention at C1q could preempt tagging by the classical pathway and prevent loss of synaptic fate in vulnerable RGC synapses [81]. With the C3 blockade that also counteracts the central amplification hub, widespread opsonization is limited, and C3a-driven glial priming is quelled. Chemoattraction from C5a, and therefore C5 inhibition, intercepts the direct membrane MAC and C5a-related membrane stress. Locally delivered (intravitreal, periocular, or sustained-release) ocular therapeutics primarily target the retinal–ONH axis and minimize the risk of systemic infection [82]. The central translational paradox is the complement tagging of inflammation subprocesses that could be homeostatic–complement opportunistic, such that complement blockers are most effective during the early to mid-stages, before secondary debris accumulates to a degree that higher-order opsonization could be more harmful.

Complement activation occurs chiefly upstream of *NLRP3* inflammasome signaling in glaucoma. Local production of the anaphylatoxins C3a and C5a acts on their respective receptors on microglia and other glial cells, which triggers NF-κB–driven transcription of components of the inflammasome and of pro–IL-1β. This process subsequently primes the *NLRP3* inflammasome [67]. Its activation occurs via the reflection of intracellular endogenous danger signals, including ATP via the P2X7 receptor, mitochondrial reactive oxygen species, and erratic ionic shifts. These signals lead to the caspase-1—dependent maturation of the cytokines IL-1β and IL-18. It is noteworthy that IL-1β, which is generated in association with inflammasomes, stimulates further expression and deposition of complement components [67]. This phenomenon initiates a feed-forward inflammatory loop which perpetuates and enhances neuroinflammation and the associated accelerated degeneration of retinal ganglion cells [83].

### 7.2. NLRP3 Inflammasome and IL-1β Axis Suppression

Therapies targeting the inflammasome aim to mitigate IL-1β/IL-18 toxicity, pyroptosis, and neuroinflammatory silencing, as well as chronic neuroinflammation [84]. *NLRP3* inhibitors and caspase-1 blockers act at the site of cytokine maturation. Upstream, ATP-activated *NLRP3* antagonism may help control the ATP-activated *NLRP3* spikes that occur during ONH strain or ischemia. Clinically, IL-1 blockade has been shown to provide effective downstream control of IL-1β, despite possible residual inflammasome activity. Over-suppression of the inflammasome could prevent the initial signaling of tissue repair [85]. This could be particularly problematic in the pre-degenerative stage. This is why these control agents should be used in individuals with high IL-1β/IL-18 levels during active disease progression.

### 7.3. Microglial State Reprogramming and Adjunct Immunoregulation

Microglia execute complement- and inflammasome-linked injury and are key in surveillance and clearance. Therefore, the discipline is shifting from wholesale depletion to state reprogramming, in which neurotoxic phagocytosis and cytokine production are tempered, while still allowing microglia to perform protective clearance and trophic support. This can include limiting CR3-mediated engulfment of live synapses; modulating microglial receptors that may enhance pain resolution, such as TREM2; and promoting a metabolically less glycolytic, more pro-inflammatory, and more anti-inflammatory state of microglia [86]. For the immune-high subsets, and as anti-cytokine modulators (e.g., *TNF*-α or IL-6 pathway antibodies), systemic immune suppression is more weakly colocated with the immune suppression. Immunoregulatory trophic factors that diminish microglial inflammasome activity and enhance neuronal resilience are provided by MSCs, and serve to further extend these approaches [87]. Together, these approaches call for combination regimens, tailored to immune endotypes and disease stage, rather than a generic anti-inflammatory approach.

### 7.4. Preclinical Versus Clinical Evidence and Translational Limitations

In vitro and animal model studies show that activation of the complement system causes loss of retinal ganglion cells and synaptic remodeling during glaucomatous neurodegeneration [88]. In cases of human glaucoma, the aqueous humor and post-mortem retinal tissue show increased levels of complement components, which supports the involvement of the immune system; however, the causal relationships and the contribution of the specific stages remain elusive [89]. Investigations have also shown that *NLRP3* inflammasome activation occurs in retinal microglia due to elevated intraocular pressure. This cellular stress activates inflammation. There is also a lack of direct evidence of activation of the inflammasome in human glaucoma, which is primarily seen through indirect markers and small cohort studies, illustrating a clear need for functional and longitudinal studies in humans [90]. As for microglial activation, there is overwhelming evidence in rodent models that microglia adopt a pro-inflammatory phenotype, which is also associated with increased neuronal damage. There is also evidence in humans that microglial markers are increased in the retinal and optic nerve head tissues; however, their functional state and the temporal dynamics have not been fully described [91]. This evidence shows the limitations and strengths of the current experimental models and shows the importance of successfully translating the immune-based discoveries to the clinical management of glaucoma.

## 8. Translation Challenges and Trial Design

Immunologically targeted, neuroprotective therapy for glaucoma has strong theoretical foundations; however, it is translationally problematic. The first challenge is that glaucoma is a syndrome, rather than a single disease. Immune pathways cross and vary in contribution across individual patients, disease stages, and disease subtypes. If clinical trials recruit broad, ‘all-comers’ populations using only intraocular pressure (IOP)-based or structural inclusion criteria, true biological responses may be masked by immune non-responder participants, and underpowered negative trials may be recorded for valid biological net responses. A second challenge is temporal: complement, inflammasome, and microglial programs can be beneficial in acute tissue repair, but when engaged chronically, they become detrimental; thus, the therapeutic window is likely stage-dependent. Finally, immune-based therapeutic interventions require robust, convincing evidence that the therapeutic target has been engaged in the eye, and this is difficult to demonstrate without validated pharmacodynamic metrics and endpoints that are sensitive to neuroprotection within clinically meaningful timeframes. These challenges can be overcome using a mechanistic approach in clinical trial design, which may help us determine relevant participant populations and appropriate target order for multimodal endpoints that describe early neuronal restoration and delivery mechanisms that fine-tune a clinically relevant balance between ocular specificity and systemic safety.

### 8.1. Disease Heterogeneity and Identification of Responders

Glaucoma is heterogeneous in its presentation as primary open-angle, normal-tension, angle-closure, and secondary forms, all of which will have different biomechanical, vascular, and immunological settings [92]. There may be additional immune variations in different subtypes within each of the forms based on age, genetics, systemic inflammation and other comorbidities, resulting in the varied immune phenotypes such as complement-dominant, inflammasome-dominant, microglia-dominant, mixed immune-metabolic or immune-low. The trials which are conducted without any subtypes or without any prioritization of responders will likely suffer from responder dilution. Some of the immunological fluid panels (complement fragments, *IL-1β/IL-18*, *TNF*-α), glia activation rather immunological markers and imaging systems, and subsequent targeted subgroup analyses are viable solutions. As more information is compiled, adaptive and platform trial designs are likely the most beneficial, enabling realignment of therapeutics towards biomarker-defined responders.

### 8.2. Endpoints Appropriate for Immune Therapies

Since immune therapies do not directly impact IOP, endpoints need to directly assess neurodegeneration instead. Longitudinal OCT metrics and visual field progression rates (VPs) are still central to the field; however, the sensitivity of these metrics may be limited in shorter studies. Functional RGC (Retinal Ganglion Cell) readouts such as posterior photopic ERG PRN and PERG may be able to detect earlier rescue effects and could also be considered as intermediate efficacy endpoints [93]. It is also important to determine the pharmacodynamic active engagement of immune candidates such as shifts in the aqueous IL-1β and complement levels, inflammasome-related proteins, and proxies of inflammation that are modulated to suggest imaging inflammation [94]. The combination of all three structural, functional, and biomarker endpoints is likely to improve the estimation of the true effect and is also likely to improve the interpretation of the true causality.

### 8.3. Delivery Routes, Dosing Windows, and Safety Tradeoffs

Delivering the product is the most critical and translational decision, considering the immune pathways that connect the eye and body [95]. Local routes (intravitreal injection, periocular delivery, sustained-release implants) maximize retinal/ONH exposure and minimize systemic immunosuppression, but at the cost of adding procedural burden and the need for careful infection, hemorrhage, or retinal toxicity monitoring. While systemic routes can be much easier for patients and may even be beneficial for patients with strong peripheral priming, they bring the trade-offs of off-target immune effects and long-term safety issues. Hybrid strategies targeted at nanoparticles, antibody fragments with ocular tropism, or pro-drugs activated in ocular tissues may reduce these tradeoffs. Regardless of the route, dosing needs to be calibrated to the disease stage and biomarker activity to target maladaptive chronic immunity while preserving early immunity protective mechanisms [96].

### 8.4. Challenges and Limitations of Immune-Based Therapeutic Strategies

Despite increasing interest in immune-based precision therapies for glaucoma, several challenges limit their clinical translation [97]. Modulation of immune pathways in chronic neurodegenerative diseases carries inherent risks, including incomplete therapeutic efficacy, potential adverse effects associated with long-term immune intervention, and difficulties in achieving pathway- or cell-specific targeting [98]. In addition, glaucoma is a heterogeneous disease in which immune mechanisms may vary across disease stages and patient subgroups, complicating treatment selection and outcome prediction. These considerations underscore the importance of biomarker-guided patient stratification, localized delivery approaches, and stage-appropriate intervention strategies to maximize therapeutic benefit while minimizing risk [99]. Future therapeutic success will likely depend on the integration of validated immune biomarkers with disease-stage-specific intervention strategies to enable safe and effective precision treatment in glaucoma [100].

## 9. Future Directions

New potential directions for this research involve continuing to build upon this mechanistic framework in the hope of clinical application of this research in the field of precision immunology. Foremost among these will be the high-resolution characterization of immune endotypes in human glaucoma. This will require single-cell transcriptomics, spatial proteomics, and multi-omics characterization of the human retina and optic nerve head, across different stages of the disease and subtypes, to enable the mapping of microglial, astrocyte, complement, and inflammasome states that are relevant to human patients rather than being extrapolated from animal studies. These data should be cross-correlated with clinical micro-phenotyping to determine which immune programs correspond to accelerated disease course, normal-tension glaucoma, or treatment resistance. Another main pillar will be the thorough and systematic validation of potential biomarkers. Candidate biomarkers should be tested iteratively and prospectively in large data sets to determine generalizability, stage predictivity, and sensitivity. There is also a need for biomarkers to serve as pharmacological readouts for potential immunomodulators, providing evidence of target engagement in trials before significant changes in structure can be observed. There is also a high likelihood that future treatment approaches will operate using a rationally designed strategy that combines immune modulators with metabolic and vascular stressors. Finally, large-scale deployment of these approaches will need to employ a high level of computational integration.

Machine learning algorithms that merge immune biomarkers, OCT/visual field progression, and vascular and genetic data can provide customized estimates of progression and personalized predictions of matched therapy. If these priorities are addressed, the field of glaucoma will be able to pivot from damage control of advanced stages to proactive endotype-specific neuroprotection.

## 10. Conclusions

This review identifies the complement–*NLRP3*–microglia axis as the primary model of neurodegeneration of glaucoma from the immune system. Complement activation, the signaling of the inflammasome, and microglial effector responses are not isolated. The integration of the three to form a persistent inflammatory network, even when the stimuli have been removed, leads to further damage of tissues. The balance of metabolic and systemic immune factors in the complement–*NLRP3*–microglia model gives the justification for the design of sophisticated immune-modulatory and neuroprotective strategies to treat glaucoma. A combined feedback loop of complement activation and shifts in the *NLRP3* inflammasome, and microglial states, drives synaptopathy, axonal damage, and the death of RGCs, which are far too often independent of IOP. Along the pathway, complement-tagged synapses and cells are lost, and danger signals are synthesized into injurious IL-1β/IL-18, and microglia are brought in to amplify the response. This pathway explains why some patients progress despite pressure control and suggests immune mechanisms as traditionally unrecognized risk factors. The combination of immune mechanisms and clinical stratification of patients to target complement, inflammasome, and microglial control will likely provide a paradigm shift in glaucoma management to an individualized, neuroprotective approach.

## Figures and Tables

**Table 1:** Mechanistic Summary of the Complement–*NLRP3*–Microglia Axis.

Component	Major Glaucoma-Relevant Triggers	Key Effectors Mediators	Primary Cellular Targets	Core Pathogenic Consequences	References
Complement system (C1q–C3–C5)	RGC metabolic stress; optic nerve head (ONH) biomechanical strain; oxidative stress (ROS); mitochondrial dysfunction; late apoptosis; reactive astrocytes	C3b/iC3b opsonins; C3a and C5a anaphylatoxins; sublytic membrane attack complex (MAC, C5b-9)	RGC synapses and dendrites; RGC membranes; microglia (via CR3); astrocytes	Aberrant synaptic tagging and pruning; phagoptosis of stressed but viable neurons; glial priming; oxidative and membrane stress	[32,61]
*NLRP3* inflammasome	ATP–P2X7 signaling; mitochondrial ROS and oxidized mtDNA; K^+^ efflux; hypoxia/ischemia; lysosomal stress; DAMPs; complement-mediated priming	Caspase-1 activation; IL-1β and IL-18 maturation; gasdermin-mediated pore formation; pyroptosis	Microglia; astrocytes; ONH glial and vascular cells; stressed RGCs	Chronic cytokine toxicity; disruption of retinal and ONH barriers; impaired axonal transport; inflammatory cell death of neurons and glia	[62,63]
Microglia (state-dependent effector cells)	Complement opsonization (C1q/C3b); C3a/C5a signaling; TLR ligands; IL-1β/*TNF*-α; mechanical strain; hypoxic and metabolic stress	CR3-mediated phagocytosis; cytokines (IL-1β, *TNF*-α); ROS/RNS; antigen presentation (MHC-II); local complement production	RGC synapses and somas; astrocytes and Müller glia; vascular and barrier interfaces	Early debris clearance and repair → late maladaptive synapse loss, axonal dysfunction, and RGC degeneration	[64,65]
Feed-forward circuit coupling	Complement anaphylatoxins (C3a/C5a) priming inflammasomes; IL-1β–driven complement induction; DAMP release from phagocytosis and pyroptosis	Self-reinforcing complement–inflammasome–microglia amplification loop	Retina and ONH immune microenvironment	Sustained neuroinflammation and progression despite adequate intraocular pressure control	[66,67]

**Table 2:** Immune-Linked Biomarkers and Mechanism-Matched Therapies for Glaucoma.

Stratification Feature	Suggested Biomarkers	Dominant Mechanism	Therapy Candidates	Translation Notes
Complement-high early neurodegeneration	Aqueous C1q/C3a/C5a; retinal C3 deposition proxies	Complement-microglia synaptopathy	C1q/C3/C5 inhibitors; CR3-phagocytosis modulators	Best window likely early or mid-disease
Inflammasome-high inflammation	IL-1β/IL-18; *NLRP3*/ASC transcripts; P2X7 activity	*NLRP3*-microglia cytokine toxicity	*NLRP3* inhibitors; caspase-1 blockers; IL-1R antagonists	Stage-aware dosing required
Microglial maladaptive state	OCT hyper-reflective foci; TSPO-PET (research); microglial gene signatures	Microglia-driven phagoptosis/ROS	Microglial reprogrammers; TREM2 agonism; partial CSF1R modulation	Avoid global ablation
Systemic immune/autoimmune subset	HSP antibodies; serum *TNF-α/IL-6*; complement activity	Systemic priming of retinal immunity	Systemic biologics; tolerogenic strategies	Likely smaller cohort, needs selection
Mixed immune-metabolic glaucoma	Immune panel + mitochondrial/oxidative markers	Coupled immune-metabolic degeneration	Combination immunomodulation and vascular/mito support	Probably most common phenotype

## Data Availability

No new data were created or analyzed in this study.
